# Curiosity About and Susceptibility Toward Hookah Smoking Among Middle and High School Students

**DOI:** 10.5888/pcd16.180288

**Published:** 2019-01-10

**Authors:** Andrea S. Gentzke, Baoguang Wang, Joelle N. Robinson, Elyse Phillips, Brian A. King

**Affiliations:** 1 Office on Smoking and Health, National Center for Chronic Disease Prevention and Health Promotion, Centers for Disease Control and Prevention, Atlanta, Georgia; 2 Center for Tobacco Products, US Food and Drug Administration, Silver Spring, Maryland

## Abstract

**Introduction:**

Hookah smoking has increased among young people. Curiosity and susceptibility may be associated with experimentation or established use. Because tobacco use behaviors are established primarily during adolescence, our objective was to examine factors that may increase the risk of future tobacco product use among youth.

**Methods:**

We analyzed data from the 2016 National Youth Tobacco Survey, a nationally representative survey of US students. Analyses were restricted to youth who had never smoked a hookah and stratified by their ever having used other tobacco products. The prevalence of hookah curiosity and susceptibility was assessed by sociodemographic characteristics, perceptions of harmfulness and addictiveness of hookahs, and peer use of hookahs. Associations between covariates and curiosity and susceptibility were assessed by using multivariable-adjusted regression.

**Results:**

Overall, 29.1% of students reported any hookah curiosity or susceptibility. Curiosity was reported by 14.6% of those who never used tobacco products and by 45.9% of those who ever used tobacco products. Hookah susceptibility was reported by 15.6% of never-users and 52.5% of ever-users. Regardless of ever having used other tobacco products, odds of curiosity and susceptibility were higher among students with perceptions of reduced hookah harmfulness and addictiveness and among those who perceived high levels of hookah use among peers.

**Conclusion:**

Nearly 3 in 10 youth who never smoked a hookah (6.9 million) reported hookah curiosity or susceptibility, and prevalence was highest among those who had ever used other tobacco products. These findings reinforce the importance of educating youth about the dangers of all tobacco products and dispel misperceptions about the harmfulness and addictiveness of hookah smoking. Continued surveillance of youth curiosity, susceptibility, and use of hookahs can inform public health policy and practice.

## Introduction

Tobacco use behaviors are established primarily during adolescence (1). Although population-based tobacco control interventions have contributed to significant declines in youth cigarette smoking (1), the diversification of the tobacco product landscape has contributed to changes in patterns of tobacco product use among US youth. Since 2011, use of novel tobacco products, such as hookahs and electronic cigarettes, has increased, offsetting declines in use of conventional tobacco products such as cigarettes. As a result, no overall change in tobacco product use among middle and high school students has occurred (2). In 2016, 4.8% of high school students reported current (in the past 30 days) hookah smoking (2).

Adolescence is a critical period of brain development in impulse control and reward-seeking systems (3). Thus, this period is an ideal time to monitor factors that are associated with experimentation or initiation of tobacco use, such as curiosity and susceptibility (4–7). Curiosity refers to an interest in tobacco products, even in the absence of intention to use tobacco (5). Susceptibility is the development of beliefs about future tobacco use behaviors, which may inform the likelihood of experimentation or establishment of use (4). The presence of curiosity or susceptibility may identify adolescents who may progress from nonuse to experimentation or established tobacco use (4,6,7).

Few studies have assessed curiosity about hookah smoking or susceptibility to it. Existing studies focusing on young adults or college-age populations suggest that people susceptible to hookah-smoking are more likely to be male, use other tobacco products, have reduced perceptions of the harmfulness and addictiveness of hookah smoking, and believe that smoking a hookah is socially acceptable (8–10). However, among current young adult hookah smokers, the median age of initiation is 17.4 years (11). Because curiosity and susceptibility are precursors to tobacco use (4,6), it is important to monitor these constructs among youth. We examined the prevalence and correlates of hookah curiosity and susceptibility in 2016 among a nationally representative sample of US middle and high school students who never smoked a hookah.

## Methods

### Data source

We used data from the 2016 National Youth Tobacco Survey (NYTS), a cross-sectional, paper-and-pencil survey administered to a nationally representative sample of US students in grades 6 through 12. NYTS uses a 3-stage cluster sampling design to provide representative estimates of US students attending public and private schools. In 2016, 20,675 students participated in NYTS (overall response rate, 71.6%). We used secondary de-identified public use data in our analysis and therefore did not require human subject review.

Because curiosity and susceptibility are precursors to experimentation or established tobacco use (4,6), we restricted our analytic sample to youth who had never smoked a hookah (never smoker) on the basis of an answer of no to the question, “Have you ever tried smoking tobacco in a hookah or water pipe, even 1 or 2 puffs?” (n = 17,846).

### Measures

To assess curiosity, respondents were asked, “Have you ever been curious about smoking tobacco in a hookah or water pipe?” Response options were: “definitely yes,” “probably yes,” “probably not,” and “definitely not.” Consistent with previous literature (5,6), we categorized curiosity as highly curious (definitely yes, probably yes), somewhat curious (probably not), or not curious (definitely not). Literature indicates that respondents answering probably not are at greater risk of smoking than respondents answering definitely not (6); thus, the 2 responses remained separate categories (somewhat curious and not curious). This marker is validated as a predictor of smoking initiation (6). Respondents with missing data (n = 35) were excluded from the analysis.

Susceptibility was assessed by 3 questions: “Do you think you will try smoking tobacco in a hookah or water pipe soon?,” “Do you think you will smoke tobacco in a hookah or water pipe in the next year?,” and “If one of your best friends offered you a hookah or water pipe with tobacco, would you try it?” Response options for each question were definitely yes, probably yes, probably not, and definitely not. These questions were combined into a susceptibility index, which has been validated as a predictor of smoking initiation (4). Tobacco susceptibility measures the lack of a firm commitment not to use tobacco products (12). Therefore, susceptibility was defined as a response of definitely yes, probably yes, or probably not to at least 1 question (13). Respondents answering definitely not to all questions were defined as not susceptible. Respondents with missing data for all 3 questions (n = 17) were excluded from the index and analyses.

Study covariates were sex (male, female), race/ethnicity (non-Hispanic white, non-Hispanic black, Hispanic, non-Hispanic other), school level (middle school [grades 6–8], high school [grades 9–12]), current (past 30 days) use of other tobacco products (cigarettes; e-cigarettes; cigars, little cigars, or cigarillos; smokeless tobacco; pipe; bidis) (no, yes), and current tobacco product use by a household member (no, yes). Hookah curiosity and susceptibility were also assessed by perceptions of hookah harmfulness (no/little harm, some harm, a lot of harm), relative hookah addictiveness compared with cigarettes (less addictive, equally addictive, more addictive, or don’t know), peer hookah use (low [≤20% of peers], high [>20% of peers]), and overall tobacco product harm (strongly agree/agree vs strongly disagree/disagree that all tobacco products are dangerous). NYTS data and methods, including the 2016 survey, are available elsewhere (14).

### Analysis

Analyses were completed by using SAS-callable SUDAAN version 11 (RTI International) to account for the complex sampling design and were weighted to provide nationally representative estimates. Youth who have ever used tobacco may be more likely to show interest in trying additional tobacco products (15). Thus, all analyses were completed separately for ever users and never users of other tobacco products. Prevalence estimates with 95% confidence intervals (CIs) of hookah curiosity and susceptibility were assessed overall and for each covariate; significant differences across levels of each covariate were assessed by χ^2^ test (*P* < .05). Weighted population counts of curiosity and susceptibility were estimated from extrapolated probability weights; reported counts were rounded down to the nearest 10,000 persons.

The association between hookah curiosity and each covariate was assessed by using multinomial logistic regression. Binary logistic regression examined the association between hookah susceptibility and each covariate. Adjusted odds ratios (aORs) and 95% CIs were computed.

## Results

Of the 17,846 youth who never smoked a hookah (Table 1), 52.9% were in high school, 50.4% were male, 57.4% were non-Hispanic white, 25.2% had ever tried another tobacco product, and 8.3% were current users of another tobacco product.

**Table 1:** Sociodemographic Characteristics of US Middle And High School Students Who Never Smoked a Hookah, Overall and by Use of Other Tobacco Products, National Youth Tobacco Survey, 2016^a^

Characteristic	Overall	Never Used Tobacco Product	Ever Used Tobacco Product
**Overall no.**	17,846	13,469 (74.8) [73.1–76.5]	4,377 (25.2) [23.5–26.9]
**School level**			
Middle school	8,893 (47.1) [41.5–52.7]	7,536 (53.4) [47.5–59.3]	1,357 (28.6) [23.1–34.0]
High school	8,892 (52.9) [47.3–58.5]	5,886 (46.6) [40.7–52.5]	3,006 (71.4) [66.0–76.9]
**Sex**			
Male	8,963 (50.4) [49.4–51.4]	6,585 (49.1) [48.0–50.1]	2,378 (54.4) [52.2–56.6]
Female	8,785 (49.6) [48.6–50.6]	6,813 (50.9) [49.9–52.0]	1,972 (45.6) [43.4–47.8]
**Race/ethnicity**			
Non-Hispanic white	7,996 (57.4) [53.1–61.7]	6,017 (57.4) [53.0–61.8]	1,979 (57.4) [52.5–62.2]
Non-Hispanic black	2,770 (12.9) [10.1–15.8]	2,055 (12.9) [10.0–15.7]	715 (13.2) [10.0–16.4]
Hispanic	4,755 (24.0) [20.9–27.1]	3,491 (23.4) [20.4–26.5]	1,264 (25.7) [21.8–29.7]
Non-Hispanic other	1,496 (5.6) [4.5–6.8]	1,233 (6.3) [5.0–7.6]	263 (3.7) [2.8–4.6]
**Household member uses any tobacco product**			
No	11,099 (65.2) [62.8–67.7]	9,134 (71.1) [68.8–73.4]	1,965 (47.7) [44.9–50.5]
Yes	5,760 (34.8) [32.3–37.2]	3,634 (28.9) [26.6–31.2]	2,126 (52.3) [49.5–55.1]
**Perception of hookah harm **			
None/little harm	2,661 (14.8) [14.0–15.7]	1,659 (12.0) [11.1–12.8]	1,002 (23.4) [21.5–25.4]
Some harm	6,156 (36.3) [34.8–37.8]	4,464 (34.9) [33.2–36.5]	1,692 (40.4) [38.2–42.7]
A lot of harm	8,513 (48.9) [47.4–50.4]	6,980 (53.2) [51.6–54.8]	1,533 (36.1) [33.7–38.6]
**Perception of tobacco harm**			
Agree that all tobacco products are dangerous	15,811 (91.5) [90.8–92.3]	12,207 (93.4) [92.8–94.0]	3,604 (86.0) [84.4–87.6]
Disagree that all tobacco products are dangerous	1,528 (8.5) [7.7–9.2]	913 (6.6) [6.0–7.2]	615 (14.0) [12.4–15.6]
**Perception of hookah addictiveness versus cigarettes**			
Equally addictive	5,359 (32.5) [31.0–34.0]	3,815 (30.7) [29.0–32.4]	1,544 (37.7) [35.5–39.8]
Less addictive	1,763 (10.1) [9.4–10.7]	1,040 (7.7) [7.0–8.4]	723 (17.1) [15.4–18.9]
More addictive	2,072 (11.5) 10.6–12.3]	1,458 (10.7) [9.9–11.6]	614 (13.6) [12.1–15.1]
Don't know enough about product(s)	8,122 (46.0) [44.5–47.6]	6,777 (50.8) [49.1–52.6]	1,345 (31.6) [29.7–33.5]
**Perception of peer hookah smoking^b^**			
Low use (≤20%)	11,025 (66.4) [63.7–69.2]	8,669 (68.9) [66.1–71.8]	2,356 (59.1) [56.2–62.1]
High use (>20%)	6,183 (33.6) [30.8–36.3]	4,331 (31.1) [29.2–33.9]	1,852 (40.9) [39.9–43.8]
**Current use of any tobacco product^c^**			
No	16,484 (91.7) [90.8–92.7]	—c	3,029 (67.2) [64.9–69.6]
Yes	1,349 (8.3) [7.3–9.2]	—c	1,347 (32.8) [30.4–35.1]

a Respondents with missing data on use of other tobacco products (n = 52) were excluded. Values are number (percentage) [95% confidence interval]. Number is based on unweighted data; percentage and 95% confidence intervals are based on weighted data. Numbers may not match totals because of missing data; percentages may not total 100% because of rounding.

b Refers to perception of how much peers use tobacco. Response options ranged from 0 to 10. A cut point of 20% was the median.

c Among ever tobacco product users, only (n = 4,733). Estimate is not applicable for respondents who had never used a tobacco product.

### Curiosity

**Students who never used tobacco products.** Among students who had never used tobacco products, 14.6% were curious about hookah smoking; 6.2% (1.1 million) were highly curious about hookah smoking, and 8.4% (1.5 million) were somewhat curious (Table 2). A greater proportion of high school students (7.9%) than middle school students (4.7%) reported being highly curious about hookah smoking as did more girls (7.9%) than boys (4.4%) and more Hispanic students (9.1%) and non-Hispanic black students (9.0%) than non-Hispanic white students (4.3%). The prevalence of being highly curious was greatest among students who perceived hookah smoking as less addictive than cigarettes (20.6%) or as causing little or no harm (17.8%), or who perceived that more than 20% of their peers smoked a hookah (11.1%). Students who disagreed that all tobacco products are dangerous had an increased prevalence of high hookah curiosity (12.8%).

**Table 2:** Estimated Prevalence of Hookah Curiosity^a^ and Susceptibility^b^ Among US Middle and High School Students (N = 17,846), by Sociodemographic Characteristic, National Youth Tobacco Survey, 2016

Variable	Highly Curious		Somewhat Curious		Susceptible	
	% (95% CI)	Population^c^	% (95% CI)	Population^c^	% (95% CI)	Population^c^
**Never Used Tobacco Products (n = 13,469)^d^**						
**Overall^e^**	6.2 (5.5–6.9)	1,090,000	8.4 (7.7–9.1)	1,470,000	15.6 (14.5–16.7)	2,750,000
**Sex**						
Male	4.4 (3.7–5.1)^f^	370,000	7.8 (7.0–8.8)	670,000	13.2 (12.2–14.3)^f^	1,130,000
Female	7.9 (7.0–9.0)	700,000	8.9 (8.1–9.9)	800,000	17.9 (16.3–19.6)	1,600,000
**Race/ethnicity**						
Non-Hispanic white	4.3 (3.6–5.1)^f^	410,000	7.2 (6.3–8.2)	690,000	12.5 (11.3–13.9)^f^	1,210,000
Non-Hispanic black	9.0 (7.8–10.4)	190,000	7.4 (6.2–8.9)	160,000	19.1 (16.6–22.0)	410,000
Hispanic	9.1 (7.7–10.9)	360,000	11.7 (10.5–13.1)	460,000	22.5 (20.3–24.8)	880,000
Non-Hispanic other	6.8 (5.1–9.0)	70,000	9.9 (8.1–12.1)	100,000	13.7 (11.3–16.5)	140,000
**School level**						
Middle school	4.7 (3.9–5.5)^f^	430,000	7.1 (6.2–8.0)	660,000	13.0 (11.6–14.6)^f^	1,220,000
High school	7.9 (7.0–8.9)	640,000	9.8 (8.8–11.0)	800,000	18.5 (17.0–20.0)	1,510,000
**Household member uses any tobacco product**						
No	5.9 (5.2–6.7)	700,000	8.4 (7.7–9.2)	1,000,000	14.5 (13.4–15.7)^f^	1,730,000
Yes	7.4 (6.1–9.0)	350,000	9.1 (7.9–10.4)	440,000	19.1 (16.9–21.4)	920,000
**Perception of hookah harm **						
None/little harm	17.8 (15.8–20.1)^f^	360,000	16.3 (13.6–19.4)	330,000	37.3 (33.6–41.2)^f^	760,000
Some harm	7.0 (5.9–8.2)	410,000	11.1 (9.9–12.5)	660,000	19.7 (18.0–21.4)	1,180,000
A lot or harm	3.2 (2.7–3.8)	290,000	5.0 (4.4–5.6)	450,000	8.3 (7.4–9.3)	750,000
**Perception of tobacco harm**						
Agree that all tobacco products are dangerous	5.7 (5.0–6.5)^f^	920,000	8.2 (7.6–9.0)	1,320,000	14.7 (13.6–16.0)^f^	2,370,000
Disagree that all tobacco products are dangerous	12.8 (10.2–16.1)	140,000	11.5 (8.8–14.9)	130,000	27.8 (23.9–32.2)	310,000
**Perception of hookah addictiveness versus cigarettes**						
Equally addictive	6.5 (5.4–7.7)^f^	340,000	8.5 (7.8–9.2)	1,450,000	17.8 (16.3–19.5)^f^	940,000
Less addictive	20.6 (17.3–24.3)	270,000	10.0 (8.8–11.3)	520,000	37.9 (33.6–42.4)	500,000
More addictive	8.3 (6.1–11.3)	150,000	16.8 (14.2–19.9)	220,000	17.8 (15.2–20.9)	330,000
Don't know enough about product(s)	3.5 (3.0–4.2)	300,000	9.2 (7.4–11.4)	170,000	10.6 (9.5–11.9)	930,000
**Perception of peer hookah smoking^g^**						
Low use (≤20%)	4.0 (3.5–4.7)^f^	470,000	7.1 (6.4–7.8)	830,000	12.0 (11.0–13.2)^f^	1,410,000
High use (>20%)	11.1 (9.8–12.6)	580,000	11.8 (10.5–13.2)	620,000	24.1 (22.4–25.9)	1,270,000
**Ever Used Tobacco Products (n = 4,377)^d^**						
**Overall**	25.7 (23.7–27.8)	1,520,000	20.2 (18.5–22.0)	1,190,000	52.5 (50.5–54.6)	3,120,000
**Sex**						
Male	22.1 (19.3–25.1)^f^	700,000	21.5 (19.6–23.6)	690,000	50.0 (47.5–52.5)^f^	1,600,000
Female	29.9 (27.5–32.5)	800,000	18.7 (16.4–21.2)	500,000	55.6 (52.3–58.8)	1,490,000
**Race/ethnicity**						
Non-Hispanic white	25.3 (22.7–28.1)^f^	830,000	20.5 (18.2–23.1)	670,000	53.5 (50.4–56.5)^f^	1,750,000
Non-Hispanic black	27.4 (21.7–33.8)	200,000	12.8 (9.6–16.9)	90,000	43.2 (39.4–47.1)	320,000
Hispanic	26.9 (23.8–30.1)	390,000	23.6 (21.2–26.2)	340,000	56.4 (53.2–59.6)	830,000
Non-Hispanic other	28.1 (21.6–35.8)	50,000	—h	—h	50.7 (41.7–59.7)	100,000
**School level**						
Middle school	22.5 (18.5–27.2)^f^	380,000	19.2 (17.1–21.4)	320,000	47.6 (44.4–50.8)^f^	800,000
High school	26.9 (24.8–29.1)	1,130,000	20.6 (18.5–23.0)	870,000	54.4 (52.1–56.7)	2,300,000
**Household member uses any tobacco product**						
No	25.2 (22.6–28.0)	670,000	21.2 (19.0–23.5)	560,000	53.2 (50.7–55.8)	1,420,000
Yes	26.1 (23.6–28.7)	760,000	19.3 (17.2–21.7)	60,000	52.0 (49.3–54.8)	1,520,000
**Perception of hookah harm**						
No/little harm	42.3 (38.2–46.5)^f^	570,000	22.9 (19.8–26.3)	300,000	72.2 (68.7–75.4)^f^	970,000
Some harm	23.8 (21.2–26.5)	550,000	24.0 (20.9–27.5)	560,000	58.7 (55.7–61.6)	1,360,000
A lot of harm	17.2 (14.5–20.4)	350,000	14.1 (12.0–16.5)	290,000	33.3 (30.2–36.6)	690,000
**Perception of tobacco harm**						
Agree “all tobacco products are dangerous”	24.8 (22.9–26.7)^f^	1,220,000	19.6 (17.8–21.6)	970,000	51.1 (49.0–53.3)^f^	2,530,000
Disagree “all tobacco products are dangerous”	30.5 (26.6–34.7)	240,000	23.3 (20.2–26.7)	180,000	61.5 (56.3–66.4)	490,000
**Perception of hookah addictiveness versus cigarettes**						
Equally addictive	27.6 (25.1–30.3)^f^	590,000	21.9 (19.3–24.8)	470,000	57.5 (54.2–60.8)^f^	1,250,000
Less addictive	41.0 (35.7–46.4)	400,000	22.3 (18.6–26.5)	220,000	69.7 (65.6–73.5)	680,000
More addictive	18.3 (14.9–22.4)	140,000	20.3 (16.3–25.0)	150,000	46.7 (42.3–51.2)	360,000
Don't know enough about product(s)	18.1 (15.3–21.3)	330,000	17.1 (16.3–25.0)	310,000	40.1 (36.5–43.8)	730,000
**Perception of peer hookah use^g^**						
Low use (≤20%)	21.9 (19.2–24.8)^f^	740,000	19.5 (17.5–21.7)	660,000	48.3 (45.5–51.1)^f^	1,640,000
High use (>20%)	31.1 (28.5–34.0)	730,000	21.3 (19.0–23.7)	500,000	58.8 (56.3–61.3)	1,380,000
**Current use, any tobacco product^i^**						
No	21.4 (19.2–23.6)^f^	850,000	19.1 (17.3–21.2)	760,000	46.5 (44.1–48.8)^f^	1,850,000
Yes	34.6 (31.0–38.6)	670,000	22.3 (19.5–25.3)	430,000	65.1 (61.9–68.1)	1,260,000

Abbreviation: CI, confidence interval.

a Respondents were asked, “Have you ever been curious about smoking tobacco in a hookah or water pipe?” Responses were recoded as “highly curious” (definitely yes/probably yes), “somewhat curious” (probably no), and “not curious” (definitely no); 35 respondents were excluded due to missing data.

b Respondents were asked, “Do you think that you will try smoking tobacco in a hookah or water pipe soon?”, “Do you think you will smoke tobacco in a hookah or water pipe in the next year?”, and “If one of your best friends were to offer you a hookah or water pipe with tobacco, would you try it?” Susceptibility was defined as a response other than “definitely no” to any of these questions; 17 respondents were excluded due to missing data.

c Estimated population counts rounded down to the nearest 10,000 persons.

d Stratified by never or ever having used a hookah or other tobacco products. Respondents with missing data (n = 52) were excluded.

e Analyses were restricted to participants who never used a hookah or water pipe (n = 17,846).

f Significant χ^2^ test (*P* < .05) indicates difference in level of curiosity or susceptibility across covariate subgroups. χ^2^ analyses were completed separately for never tobacco product users and ever users.

g Refers to perception of how much peers use tobacco. Response options ranged from 0 to 10. A cut point of20% was the median.

h Unstable estimate (relative standard error >30%).

i Among students who ever used tobacco product only (n = 4,733).

After adjustment for covariates, the odds of being highly curious about hookah smoking versus not curious were greater for girls (aOR, 1.71; 95% CI, 1.42–2.07) than for boys and for non-Hispanic black students (aOR, 1.75; 95% CI, 1.42–2.17), Hispanic (aOR, 2.05; 95% CI, 1.59–2.64), and non-Hispanic other race students (aOR, 1.98; 95% CI,1.40–2.81) than for non-Hispanic white students (Table 3). Greater odds of high curiosity versus no curiosity were observed among students with low perceptions of the harmfulness of hookah smoking (no/little harm: aOR, 4.95; 95% CI, 3.73–6.57; somewhat harmful: aOR, 2.17; 95% CI, 1.77–2.66) versus a lot of harm; among students who perceived hookah smoking to be less addictive than cigarettes (aOR, 2.20; 95% CI, 1.65–2.92) versus equally addictive; among students who perceived high levels of hookah smoking among their peers (aOR, 2.12; 95% CI, 1.70–2.64) versus low use, and among students who disagreed that all tobacco products are dangerous (aOR, 2.22; 95% CI, 1.54–3.21) than those who agreed.

**Table 3:** Association Between Sociodemographic Factors and Hookah Curiosity and Susceptibility Among Youth Who Never Smoked a Hookah (N = 17,846) by Use of Other Tobacco Products, National Youth Tobacco Survey, 2016^a^

Variable	Highly Curious vs Not Curious^b^^,^ c	Somewhat Curious vs Not Curious^b^^,^ c	Susceptible vs Not Susceptible^d^
**Never Used Other Tobacco Products (n = 13,469)^e^**			
**Sex**			
Male	1 [Reference]		
Female	1.71 (1.42–2.07)^f^	1.13 (0.98–1.32)	1.31 (1.16–1.47)^f^
**Race/ethnicity**			
Non-Hispanic white	1 [Reference]		
Non-Hispanic black	1.75 (1.42–2.17)^f^	0.95 (0.75–1.21)	1.39 (1.14–1.69)^f^
Hispanic	2.05 (1.59–2.64)^f^	1.69 (1.39–2.06)^f^	1.80 (1.52–2.14)^f^
Non-Hispanic other	1.98 (1.40–2.81)^f^	1.58 (1.19–2.09)^f^	1.22 (0.97–1.55)
**School level**			
Middle school	1 [Reference]		
High school	1.18 (0.93–1.51)	1.08 (0.87–1.35)	1.07 (0.90–1.27)
**Household member uses any tobacco product**			
No	1 [Reference]		
Yes	1.20 (0.96–1.48)	1.07 (0.88–1.31)	1.38 (1.16–1.65)^f^
**Hookah harm perception**			
A lot of harm	1 [Reference]		
Some harm	2.17 (1.77–2.66)^f^	2.28 (1.87–2.78)^f^	2.47 (2.10–2.90)^f^
No/little harm	4.95 (3.73–6.57)^f^	3.60 (2.64–4.92)^f^	5.00 (3.99–6.28)^f^
**Tobacco harm perception**			
Agree “all tobacco products are dangerous”	1 [Reference]		
Disagree “all tobacco products are dangerous”	2.22 (1.54–3.21)^f^	1.40 (1.04–1.89)^f^	1.94 (1.46–2.56)^f^
**Perception of hookah addictiveness versus cigarettes**			
Equally addictive	1 [Reference]		
Less addictive	2.20 (1.65–2.92)^f^	1.52 (1.22–1.88)^f^	1.48 (1.18–1.86)^f^
More addictive	1.40 (0.99–1.97)	1.12 (0.83–1.51)	1.08 (0.87–1.36)
Don't know enough about product(s)	0.53 (0.41–0.67)^f^	0.62 (0.51–0.76)^f^	0.56 (0.47–0.66)^f^
**Perception of peer hookah smoking^g^**			
Low use (0–20%)	1 [Reference]		
High use (>20%)	2.12 (1.70–2.64)^f^	1.57 (1.32–1.88)^f^	1.71 (1.53–1.91)^f^
**Ever Used Other Tobacco Products (n = 4,377)^c^**			
**Sex**			
Male	1 [Reference]		
Female	1.49 (1.17–1.90)^f^	0.99 (0.80–1.22)	1.32 (1.08–1.60)^f^
**Race/ethnicity**			
Non-Hispanic white	1 [Reference]		
Non-Hispanic black	0.97 (0.64–1.49)	0.56 (0.38–0.83)^f^	0.67 (0.53–0.85)^f^
Hispanic	1.29 (0.97–1.72)	1.35 (1.08–1.69)^f^	1.24 (1.02–1.51)^f^
Non-Hispanic other	1.57 (1.02–2.43)^f^	1.17 (0.67–2.01)	1.18 (0.76–1.84)
**School level**			
Middle school	1 [Reference]		
High school	0.99 (0.73–1.35)	1.00 (0.78–1.27)	0.97 (0.82–1.16)
**Household member uses any tobacco product**			
No	1 [Reference]		
Yes	0.94 (0.75–1.17)	0.86 (0.68–1.07)	0.86 (0.74–1.00)
**Hookah harm perception**			
A lot of harm	1 [Reference]		
Some harm	1.61 (1.28–2.02)^f^	2.19 (1.71–2.82)^f^	2.83 (2.25–3.55)^f^
No/little harm	3.77 (2.84–5.01)^f^	3.03 (2.36–3.90)^f^	4.72 (3.92–5.70)^f^
**Tobacco harm perception**			
Agree “all tobacco products are dangerous”	1 [Reference]		
Disagree “all tobacco products are dangerous”	1.26 (0.97–1.62)	1.35 (1.02–1.79)^f^	1.34 (1.09–1.64)^f^
**Perception of hookah addictiveness versus cigarettes**			
Equally addictive	1 [Reference]		
Less addictive	1.27 (0.97–1.65)	1.07 (0.77–1.47)	1.05 (0.81–1.36)
More addictive	0.61 (0.43–0.87)^f^	0.93 (0.61–1.43)	0.81 (0.62–1.06)
Don't know enough about product(s)	0.50 (0.39–0.64)^f^	0.65 (0.51–0.84)^f^	0.50 (0.40–0.63)^f^
**Perception of peer hookah use**			
Low use (0–20%)	1 [Reference]		
High use (>20%)	1.61 (1.30–2.00)^f^	1.35 (1.11–1.66)^f^	1.50 (1.29–1.73)^f^
**Current use, any tobacco product^h^**			
No	1 [Reference]		
Yes	2.34 (1.84–2.97)^f^	1.58 (1.26–1.98)^f^	2.16 (1.83–2.56)^f^

a Values are adjusted odds ratio (95% confidence interval).

b Respondents were asked, “Have you ever been curious about smoking tobacco in a hookah or water pipe?” Responses were recoded as highly curious (definitely yes/probably yes), somewhat curious (probably no), and not curious (definitely no); 35 respondents were excluded because of missing data.

c Multinomial logistic regression used to assess the association between each of these factors and curiosity; all covariates were entered into the model simultaneously. Logistic regression was used to assess the association between each factor and susceptibility; all covariates were entered into the model simultaneously.

d Respondents were asked, “Do you think that you will try smoking tobacco in a hookah or water pipe soon?”; “Do you think you will smoke tobacco in a hookah or water pipe in the next year?”; and “If one of your best friends were to offer you a hookah or water pipe with tobacco, would you try it?” Susceptibility was defined as a response other than “definitely no” to any of these questions; 17 respondents were excluded because of missing data.

e Analyses are stratified by never or ever use of other tobacco products. Respondents with missing data (n = 52) were excluded.

f Significant (*P* < .05) compared with the specified reference group for each outcome.

g Response options ranged from 0 to 10. A cut point of 20% was the median.

h Among ever tobacco product users only (n = 4,733).

**Students who ever used other tobacco products**. Among students who had ever used other tobacco products, 45.9% were curious about hookah smoking; 25.7% (1.5 million) were highly curious about hookah smoking, and 20.2% (1.2 million) were somewhat curious. High curiosity about hookah smoking was more prevalent among current users of other tobacco products (34.6%) than among those who currently did not use tobacco products (21.4%). The prevalence of being highly curious was also greater among students who perceived low harm in hookah smoking (no/little harm, 42.3%; some harm, 23.8%) than among students who perceived a lot of harm (17.2%). High curiosity was more prevalent among students who perceived hookah smoking to be less addictive than cigarette smoking (41.0%) than those who perceived it as equally addictive (27.6%) or more addictive (18.3%), and among students who perceived high use of hookah smoking among their peers (31.1%) than those who perceived low use (21.9%) (Table 2).

Greater odds of high curiosity were observed for girls (aOR, 1.49; 95% CI, 1.17–1.90), for students identifying as non-Hispanic other race/ethnicity (aOR, 1.57; CI, 1.02–2.43), for current tobacco product users (aOR, 2.34; 95% CI, 1.84–2.97), for students who perceived low harm in hookah smoking (no/little harm, aOR, 3.77, 95% CI, 2.84–5.01; some harm, aOR, 1.61, 95% CI, 1.28–2.02), and for students who perceived high hookah use among their peers (aOR, 1.61; 95% CI, 1.30–2.00). Low odds of high curiosity were observed among students who perceived hookah smoking as more addictive than cigarettes (aOR, 0.61; 95% CI, 0.43–0.87) (Table 3).

### Susceptibility

**Students who never used tobacco products.** Susceptibility to hookah smoking was reported by 15.6% (2.8 million) of students who had never used tobacco products. The prevalence of susceptibility was higher among high school students (18.5%) than among middle school students (13.0%), among girls (17.9%) than among boys (13.2%), among non-Hispanic black students (19.1%) and Hispanic students (22.5%) than among non-Hispanic white students (12.5%), and among those reporting any tobacco product use by a household member (19.1%) than those who did not (14.5%). An increased prevalence of hookah susceptibility was observed among students with reduced perceptions of hookah smoking as harmful (no/little harm, 37.3%; some harm, 19.7%) versus those who perceived a lot of harm (8.3%); among those who disagreed that all tobacco products were dangerous (27.8%) versus students who agreed that all tobacco products were dangerous (14.7%); among those who believed hookah smoking was less addictive than cigarette smoking (37.9%) versus those who believed it was equally addictive (17.8%) or more addictive (17.8%); and among students who perceived a high prevalence of hookah smoking among their peers (24.1%) versus those who perceived low prevalence (12.0%) (Table 2).

Greater odds of being susceptible to hookah smoking were observed among girls (aOR, 1.31, 95% CI, 1.16–1.47), among Hispanic students (aOR, 1.80, 95% CI, 1.52–2.14) and non-Hispanic black students (aOR, 1.39; 95% CI, 1.14–1.69), and among those reporting any tobacco product use by a household member (aOR, 1.38, 95% CI, 1.16–1.65). Increased odds of susceptibility to hookah smoking were associated with perceptions of low harm from hookah smoking (no/little harm: aOR, 5.00, 95% CI, 3.99–6.28; some harm: aOR, 2.47, 95% CI, 2.10–2.90); perception of lower addictiveness of hookah smoking than cigarette smoking (aOR, 1.48; 95% CI, 1.18–1.86); among students who disagreed that all tobacco products were dangerous (aOR, 1.94; CI, 1.46–2.56); and students who perceived high levels of hookah smoking among their peers (aOR, 1.71; 95% CI, 1.53–1.91) (Table 3).

**Students who ever used tobacco products.** Among students who ever used tobacco products, 52.5% (3.1 million) reported being susceptible to hookah smoking (Table 2). Susceptibility was greater among high school students (54.4%) than among middle school students (47.6%); among non-Hispanic white students (53.5%) and Hispanic students (56.4%) than among non-Hispanic black students (43.2%); and among current users of tobacco products (65.1%) than among nonusers (46.5%). A higher prevalence of susceptibility to hookah smoking was observed among students with low perceptions of hookah smoking as harmful (no/little harm: 72.2%, some harm: 58.7%) versus those who perceived it as harmful (a lot of harm, 33.3%); among students who perceived hookah smoking as less addictive than cigarette smoking (69.7%) versus those who perceived it as equally addictive (57.5%) or more addictive (46.7%); among students who disagreed that all tobacco products were dangerous (61.5%) than among those who agreed they were harmful (51.1%); and among students who perceived high levels of hookah smoking among their peers (58.8%) versus those who perceived low levels (48.3%).

Greater odds of susceptibility to hookah smoking was observed among girls (aOR, 1.32; 95% CI, 1.08–1.60), Hispanic students (aOR, 1.24; 95% CI, 1.02–1.51), and current tobacco product users (aOR, 2.16; 95% CI, 1.83–2.56) (Table 3). Greater odds of susceptibility to hookah smoking were observed among students with reduced perceptions of harmfulness of hookah smoking (no/little harm: aOR, 4.72; 95% CI, 3.92–5.70; some harm: aOR, 2.83; 95% CI, 2.25–3.55); students who disagreed that all tobacco products were dangerous (aOR, 1.34; 95% CI, 1.09–1.64), and students who perceived high levels of hookah smoking among their peers (aOR, 1.50; 95% CI, 1.29–1.73).

### Curiosity and susceptibility combined

Overall, 18.2% (4.3 million) of students who never tried hookah smoking reported being both curious (somewhat or highly) and susceptible to hookah smoking. Any curiosity or any susceptibility was reported by 29.1% (6.9 million) of students overall, 19.6% (3.5 million) of students who had never used tobacco products, and 57.5% (3.4 million) of students who had ever used tobacco products users.

## Discussion

Findings from our study show that nearly 7 million US middle and high school students who never smoked a hookah reported being curious about or susceptible to hookah smoking in 2016; among these students, approximately half (3.5 million) reported no previous use of any other tobacco products. Curiosity and susceptibility are factors that increase the risk of experimentation or progression to established tobacco product use (4,6,7). Continued efforts to monitor youth curiosity and susceptibility toward hookah smoking and other tobacco product use could inform public health policy, planning, and practice.

We observed that ever and current users of other tobacco products reported a greater prevalence of hookah curiosity and susceptibility than did never and noncurrent users. Youth who have experimented with tobacco products may have more interest in trying additional products (15); about half of youth who currently use tobacco products have reported using more than 1 product in the past 30 days (2). The high prevalence of curiosity about hookah smoking and susceptibility to it among youth who reported current tobacco product use is concerning, because the risk of nicotine dependence increases among users of multiple tobacco products (16). Furthermore, chronic nicotine exposure during adolescence can have lasting effects on various aspects of brain development, including attention and cognition (3). These findings underscore the importance of tobacco control campaigns aimed at educating youth about the dangers of all forms of tobacco product use.

Variation in hookah curiosity and susceptibility was observed across population subgroups. The prevalence of hookah curiosity and susceptibility was significantly higher among girls than among boys overall, although studies of the association between sex and curiosity and susceptibility to other tobacco products have yielded mixed results (17,18). Among students who never used tobacco products, the likelihood of susceptibility to hookah smoking was higher among those who indicated a household member used tobacco products than those who did not indicate tobacco product use among household members. Furthermore, among students who never used tobacco products, nonwhite racial/ethnic groups had approximately twice the odds of being highly curious or susceptible to hookah smoking. Literature suggests that initiation of tobacco product use may be delayed for nonwhite racial/ethnic groups (17), possibly resulting in differences in curiosity or susceptibility to tobacco product use among these populations. These sociodemographic variations highlight the importance of ensuring that educational interventions focused on the dangers of hookah smoking and other tobacco product use effectively reach all population groups, particularly those with the greatest burden of curiosity, susceptibility, and use.

Population-level increases in US hookah smoking have been driven largely by increased use among young people. Current hookah smoking was reported by 4.8% of high school students in 2016 (2) and by 20.1% of young adults aged 18 to 24 in 2013–2014 (19). By comparison, less than 1% of adults aged 45 to 64 reported current hookah smoking in 2013–2014 (19). Several factors may contribute to increased hookah smoking among young people. Hookah tobacco often comes in flavors that appeal more to youth (20), and these products are frequently used in social settings (11). Furthermore, there are misperceptions among young people that hookah smoking is less harmful or less addictive than cigarette smoking because of filtration or intermittent use (21). Finally, hookah smoking has greater social acceptability among young people than tobacco products (21–23).

In our study, students perceiving high levels of hookah use among their peers had higher odds of curiosity and susceptibility. Literature suggests that over half of youth perceive hookah smoking to be more socially acceptable than other tobacco products (22). This social acceptability is correlated with misperceptions of harmfulness and addictiveness; furthermore, having friends who smoke a hookah is associated with more favorable perceptions of hookah use overall (23). Evidence also suggests that most users (>90%) share hookahs with others or smoke a hookah in a social setting (11). Thus, social patterns of hookah use and peer acceptability may increase experimentation and use of hookahs.

Previous studies have reported misperceptions among youth and young adults regarding the harmfulness and addictiveness of hookah smoking (21–23). In our study, these misperceptions were associated with increased odds of curiosity and susceptibility. Before inhalation, hookah smoke passes through water and is cooled (24). This may contribute to misperceptions about filtration (24) and overall harm (23). However, hookah smoking sessions exceed 30 minutes for most users (11). During a typical smoking session, hookah users may inhale up to 100 times more smoke volume (25), 40 times more tar (26), 10 times more carbon monoxide (26), and 1.7 times more nicotine (27) than from a single cigarette. Additionally, the use of burning charcoal as a heat source places hookah users at risk for acute injury from carbon monoxide poisoning (28). Therefore, educational strategies that include messages that dispel common misperceptions about harmfulness and addictiveness of hookah smoking could be used to reduce use among youth.

Several strategies may address misperceptions and deter experimentation and hookah smoking by youth. Under authority granted by the Family Smoking Prevention and Tobacco Control Act, the US Food and Drug Administration finalized its deeming rule in 2016, extending its regulatory authorities over newly deemed products, including hookah. Hookah tobacco products now require warning statements on product packaging and advertisements and are subject to minimum age and identification requirements for purchase (29,30). States and localities can enforce requirements pertaining to all tobacco products that are more stringent than many requirements of the federal law, or in addition to federal requirements. Federal, state, and local efforts aimed at reducing hookah initiation and use among youth that could be considered are comprehensive educational campaigns on the harm and addictiveness of hookah smoking, efforts to limit youth access to hookah use, modernization of clean indoor air policies to include hookah bars and cafes, hookah-specific health warning statements, appropriate labeling of hookah tobacco packaging, or product manufacturing standards.

Our study had limitations. First, our data were collected from students enrolled in traditional middle and high schools in the United States, so our findings may not be generalizable to all US youth, particularly those who are homeschooled or not enrolled in school. Second, NYTS data are based on self-report; thus, findings may be subject to response, recall, or social desirability bias. Finally, the cross-sectional design of our study precludes the ability to assess the temporality of curiosity, susceptibility, and future hookah use.

Curiosity and susceptibility may be critical factors that increase the risk of hookah smoking among youth. Among youth who never smoked a hookah in 2016, approximately 3.5 million who never used tobacco products (19.6%) and 3.4 million who ever used tobacco product (57.5%) were curious about or susceptible to hookah smoking. These findings reinforce the importance of campaigns aimed at educating youth about the dangers of all forms of tobacco product use, particularly combustible tobacco products such as hookah. Additionally, continued surveillance of curiosity about hookah smoking and susceptibility to its use among youth can inform public health policy, planning, and practice.
